# Effect of local anesthesia with lidocaine on perioperative proinflammatory cytokine levels in plasma and cerebrospinal fluid in cerebral aneurysm patients: Study protocol for a randomized clinical trial

**DOI:** 10.1097/MD.0000000000017450

**Published:** 2019-10-18

**Authors:** Marijana Matas, Vlatka Sotošek, Ana Kozmar, Robert Likić, Ante Sekulić

**Affiliations:** a Department of Anesthesiology, Reanimatology and Intensive Care Medicine, University Hospital Center Zagreb, Zagreb,; b Department of Anesthesia, Resuscitation and Intensive Care Medicine, Faculty of Medicine, University of Rijeka,; c Department of Anesthesia and Intensive Care Medicine, Clinical Hospital Rijeka, Rijeka,; d Department of Laboratory Diagnostics, University Hospital Center Zagreb,; e Department of Internal Medicine, Unit of Clinical Pharmacology, University Hospital Center Zagreb,; f Department of Internal Medicine, University of Zagreb Medical School,; g Department of Anesthesiology, Reanimatology and Intensive Care Medicine, University of Zagreb Medical School, Zagreb, Croatia.

**Keywords:** aneurysm, cerebral, inflammation mediators, interleukins, lidocaine

## Abstract

**Background:**

Cerebral aneurysm surgery has significant mortality and morbidity rate. Inflammation plays a key role in the pathogenesis of intracranial aneurysms, their rupture, subarachnoid hemorrhage and neurologic complications. Proinflammatory cytokine level in blood and cerebrospinal fluid (CSF) is an indicator of inflammatory response. Cytokines contribute to secondary brain injury and can worsen the outcome of the treatment. Lidocaine is local anesthetic that can be applied in neurosurgery as regional anesthesia of the scalp and as topical anesthesia of the throat before direct laryngoscopy and endotracheal intubation. Besides analgesic, lidocaine has systemic anti-inflammatory and neuroprotective effect.

Primary aim of this trial is to determine the influence of local anesthesia with lidocaine on the perioperative levels of pro-inflammatory cytokines interleukin-1β, interleukin-6, and tumor necrosis factor-α in plasma and CSF in cerebral aneurysm patients.

**Methods:**

We will conduct prospective randomized clinical trial among patients undergoing craniotomy and cerebral aneurysm clipping surgery in general anesthesia. Patients included in the trial will be randomly assigned to the lidocaine group (Group L) or to the control group (Group C). Patients in Group L, following general anesthesia induction, will receive topical anesthesia of the throat before endotracheal intubation and also regional anesthesia of the scalp before Mayfield frame placement, both done with lidocaine. Patients in Group C will have general anesthesia only without any lidocaine administration. The primary outcomes are concentrations of cytokines interleukin-1β, interleukin-6 and tumor necrosis factor-α in plasma and CSF, measured at specific timepoints perioperatively. Secondary outcome is incidence of major neurological and infectious complications, as well as treatment outcome in both groups.

**Discussion:**

Results of the trial could provide insight into influence of lidocaine on local and systemic inflammatory response in cerebrovascular surgery, and might improve future anesthesia practice and treatment outcome.

**Trial is registered at ClinicalTrials.gov:**

NCT03823482.

## 1 Introduction

Cerebral aneurysms are relatively common in a general population with a prevalence of 3%. Existing treatment options include surgical and endovascular treatment, both accompanied by a significant disability and mortality rate.^[1]^ Pathophysiological mechanisms of aneurysm etiology, their growth and factors contributing to rupture with consequent bleeding are not fully known yet. Recent studies emphasize the key role of inflammation in pathogenesis of aneurysms, their rupture, subarachnoid hemorrhage (SAH) and early brain injury, spasm of cerebral vessels and delayed neurological deficit.^[2]^

The inflammatory response, measured by the increased levels of pro-inflammatory cytokines in plasma and cerebrospinal fluid (CSF), is present during and immediately after elective neurosurgical procedures, as well as in patients following cerebral aneurysm rupture.^[3–5]^ Activation of inflammatory response and cytokine release contributes to secondary brain damage and death of neurons, leading to poorer outcome. Cytokines are released from central nervous system (CNS) cells, such as microglia, astrocytes, endothelial cells of the brain and neurons, but also from activated leukocytes and lymphocytes that passed from systemic circulation through blood-brain barrier (BBB).^[6]^ Specifically, the extracellular adenosine triphosphate (ATP) released from injured brain tissue, as well as the presence of subarachnoid blood, causes proliferation, migration, and activation of the microglia. Activated microglia release various cytotoxic substances that can damage brain cells, including pro-inflammatory cytokines, interleukin (IL) 1β, tumor necrosis factor alpha (TNF-α), and interferon gamma (IFNγ). Released cytokines may pass through the damaged BBB and subsequently be detected in the blood. In addition, a systemic inflammatory response may be caused by brain injury, bleeding or ischemia.

Research show that inflammatory cytokine levels, such as IL-6 and TNF-α, in plasma and CSF of patients with aneurysmatic SAH correlate with the occurrence of neurological and infectious complications as well as with the treatment outcome.^[7,8]^

Lidocaine is local anesthetic and antiarrhythmic drug with other significant systemic effects, such as anti-inflammatory, neuroprotective and antimetastatic effects.^[9]^ In neurosurgical patient local anesthetics can be used for regional anesthesia of the scalp and before direct laryngoscopy and endotracheal intubation with the aim of maintaining hemodynamic stability and avoiding intracranial pressure increase. For this purpose, regional and intravenous administration of lidocaine are both already described.^[10]^

Lidocaine exerts significant anti-inflammatory effect. In animal research model, both inhaled and intravenous lidocaine, attenuated levels of TNF-α, IL-1β, and IL-6 in lungs.^[11]^ Another study showed that intravenous lidocaine increase the concentration of anti-inflammatory cytokine IL-10 in mechanically ventilated animal lungs.^[12]^ Clinical studies confirm that lidocaine, whether administered locally or intravenously, reduces levels of pro-inflammatory cytokines, although contradictory data exist.^[13,14]^

Recent research found significant effect of lidocaine on microglia. Lidocaine reduces the release of IL-1β and thus reduces the activation of the microglia as a key step of inflammatory response in CNS.^[15]^

The pharmacological modulation of the inflammatory process, which might be crucial in the pathology of cerebral aneurysms and treatment outcome, is still the subject of scientific research. The influence of local anesthesia with lidocaine during cerebral aneurysm surgery on inflammatory response as well as on the immediate postoperative period has so far not been systematically investigated.

We hypothesized that regional anesthesia of the scalp and topical anesthesia of the throat, both done with lidocaine, would significantly decrease the levels of pro-inflammatory cytokines in CSF and plasma of patients undergoing craniotomy and cerebral aneurysm surgery. Lower concentrations of pro-inflammatory cytokines might contribute to better outcome and might decrease the incidence of systemic and CNS related postoperative complications. Aim of this study is to determine the influence of lidocaine administration on perioperative inflammatory cytokine levels in plasma and CSF of patients undergoing craniotomy and cerebral aneurysm surgery.

## 2 Methods

### 2.1 Study design

This is an ongoing single center, prospective, randomized clinical trial at the University Hospital Center Zagreb, which is a referral hospital for cerebral aneurysm surgery and has case volume necessary for the study. Trial is registered at ClinicalTrials.gov (NCT03823482).

### 2.2 Inclusion criteria

Patient scheduled to undergo craniotomy for cerebral aneurysm surgery under general anesthesia will be recruited for the trial. Inclusion criteria include age between 18 and 70 years, American Society of Anesthesiologists (ASA) physical status I-II and written informed consent.

### 2.3 Exclusion criteria

Patients with severe cardiovascular (New York Heart Association class 3 and 4) or pulmonary condition (Global Initiative for Chronic Obstructive Lung Disease class 3 and 4) will be excluded from the trial, as well as patients with ongoing acute infection or immunological disease. Patients on steroid treatment due to any reason will also be excluded. Patients with preoperative Glasgow coma score (GCS) lower than 15 or with significant motor deficit will also be excluded. Pregnancy and allergy to any of the medication used in study protocol are also considered to be exclusion criteria. Patients with significant intraoperative blood loss (more than 500 ml) or with prolonged surgery time (more than 6 hours) will be excluded from data analysis.

### 2.4 Randomization

Patients who meet the criteria will be randomly allocated to the lidocaine group or to the control group. Randomization will be computer generated with allocation ratio 1:1. Randomization and enrollment will be done by an independent physician who is not directly involved in the care of the patients. Anesthesiologist will not be blinded to the grouping, but the participants. Unblinding could be done in case of any medical emergency. In case of unblinding, the patient will be excluded from the trial. Allocation concealment will be ensured with closed envelop method, which cannot be opened until induction of the anesthesia have been completed

### 2.5 Interventions

Patients included in the trial will be randomly assigned to the lidocaine group (Group L) or to the control group (Group C). Patients in Group L, following general anesthesia induction, will receive topical anesthesia of the throat before endotracheal intubation with 40 mg of lidocaine and also regional anesthesia of the scalp with 2% lidocaine in maximum dosage 4 mg/kg before Mayfield frame placement. Total dosage of lidocaine will be up to 400 mg. Regional anesthesia of the scalp will be performed using technique described in relevant literature.^[16]^ Patients in Group C will have general anesthesia only without any lidocaine administration. All patients will undergo craniotomy and cerebral aneurysm clipping surgery with preoperative placement of lumbar CSF drainage, when it is otherwise indicated neurosurgically.

Blood samples will be taken from every patient included in the study at four timepoints: before induction of the anesthesia (baseline), at the time of incision, at the end of surgery and 24 hours from start of anesthesia. CSF samples will be taken from lumbar CSF drainage system at 2 timepoints: at the time of incision and at the end of surgery. Concentrations of IL-1β, IL-6, and TNF-α in plasma and CSF will be measured using enzyme-linked immunosorbent assay (ELISA) specific for each cytokine, according to manufacturer instructions.

### 2.6 Anesthesia management

During the surgical procedure, the following standard monitoring will be used: electrocardiography (ECG), pulse oximetry (SpO2), invasive and noninvasive blood pressure measurements, end-tidal carbon dioxide (ETCO2), entropy, body temperature, and urine output. Induction of general anesthesia will be performed with fentanyl, propofol and rocuronium. After tracheal intubation, mechanical ventilation will be commenced to maintain normoxia and normocapnia with tidal volume of 8 to 10 ml/kg, respiratory rate of 10 to 14 per minute, fraction of inspired oxygen of 50% and fresh gas flow rate of 3 liter per minute. Anesthesia will be induced and maintained with continuous intravenous infusions of propofol 100 to 200 mcg/kg/minute and fentanyl 1 to 2 mcg/kg/hour, adjusted to entropy monitoring. No inhalation anesthetic agent will be used. Fluid balance will be closely monitored and crystalloid infusions will be used for fluid therapy. Arterial blood pressure will be continuously monitored and recorded. Vasoactive agents will be used to maintain target values of mean arterial pressure (±30% of baseline values), depending on clinical scenario, with phenylephrine 0,5 mcg/kg/minute or noradrenaline 0,01 mcg/kg/minute. Dosages of anesthetic drugs used will be recorded as well as all the monitored clinical parameters. At the end of the surgery, patient will be transferred to the intensive care unit where the patient will be safely extubated. Time of extubation will also be recorded.

Monitoring of ECG, SpO2, ETCO2, and arterial blood pressure will be continued in intensive care unit. After extubation, GCS will be recorded at every hour for the first 48 hours after surgery. Patients will be monitored for neurological and infectious complications during hospital stay and their incidence will be recorded. Any new focal motor deficit or fall in GCS by 2 points or more will be considered significant, as well as any seizure occurrence. Development of significant vasospasm will be diagnosed by clinical signs and confirmed by transcranial Doppler ultrasound (blood flow velocity more than 180 cm/seconds over middle cerebral artery). Any major pathology found on postoperative brain computerized tomography (CT) will also be recorded (bleeding, ischemia, edema, or hydrocephalus). The study will monitor for any potential intervention-related adverse events daily through patient examination and chart review. All data will be entered electronically and coded to maintain participant confidentiality. This will be done at the Center where the data originated. Original forms will be stored securely for a period of 3 years after completion of the study. All investigators will be given access to the final data sets and we will deliver a completely deidentified data set to an appropriate data archive for sharing purposes.

### 2.7 Primary outcome

The primary outcome are concentrations of IL-1β, IL-6, and TNF-α in plasma and CSF of patients undergoing craniotomy for cerebral aneurysm surgery under general anesthesia. These cytokines will be measured at specific timepoints, as described.

### 2.8 Secondary outcomes

1. Incidence of neurological complications within the first 7 postoperative days: new focal motor deficit, decrease of GCS by 2 points or more, seizure, signs of vasospasm, pathological finding in brain CT scan.
2. Incidence of infectious complications within the first 7 postoperative days: meningitis, pneumonia, or sepsis.
3. Outcome assessment will be done by using Glasgow outcome scale at the end of hospital treatment.

### 2.9 Sample size calculation

Based on previous studies, we performed a sample size estimation before recruitment with the power analysis module of STATISTICA software version 10.0 (StatSoft Inc., San Diego, CA), using α = 0.05 and β = 0.1.^[17,18]^ We determined that a minimum of 20 participants was required per group for pair-wise comparisons of our samples.

### 2.10 Timeline

This is an 18-month study, which started in March 2019 and will be ended in September 2020.

### 2.11 Safety

The incidence of side effects during entire postoperative period will be closely monitored and recorded, as well as adverse events and complications. In case of any adverse events or complications, the administration of lidocaine will be immediately stopped in Group L and patient will be excluded from further interventions of this study.

### 2.12 Ethics and dissemination

Before this study, permission was first obtained from Ethics Board of University Hospital Center Zagreb, Zagreb, Croatia (Number 02/21 AG), and Ethics Committee of Faculty of Medicine, University of Rijeka, Rijeka, Croatia (Number 217 0-24-04-3-19-3). Written informed consent will be obtained from each patient before enrollment by independent physician. Any modifications to the protocol which may impact on the conduct of the study, potential benefit of the patient or may affect patient safety, including changes of study objectives, study design, patient population, sample sizes, study procedures, or significant administrative aspects will require a formal amendment to the protocol. Such amendment will be approved by the aforementioned Ethics Committees and communicated through clinicaltrial.gov before implementation. Results of this trial will be submitted for publication in peer-reviewed journal and for presentation at scientific conference.

## 3 Statistical analysis plan

The analysis will be conducted by Statistical Package for the Social Sciences (SPSS) Statistics software, version 25.0 (IBM Corporation, Armonk, NY). Continuous and ordinal data will be presented as the median and interquartile range (IQR), while categorical data will be presented as raw numbers and as frequencies. Distribution of data will be tested by the Kolmogorov-Smirnov and Shapiro-Wilk tests. Non-parametric tests like Friedman's 2-way ANOVA with post-hoc Dunn test and Mann–Whitney *U* test will be used to assess pair-wise differences between related and independent variables with non-normal distribution. Data with normal distribution will be analyzed by Student *t* test. Correlations will be analyzed with Spearman test. For sensitivity-specificity measurements, the receiver operator characteristics (ROC) curve estimation will be used. Values of *P* < .05 is considered significant. An interim-analysis is performed on the primary endpoint when 50% of patients have been randomized. The interim-analysis is performed by an independent statistician, blinded for the treatment allocation.

## 4 Discussion

Increasing data confirm benefit of local anesthetics in patients undergoing craniotomy. Lidocaine can diminish autonomic response to the placing of Mayfield's frame when administered as scalp block or local infiltration. Regional and intravenous administration of lidocaine can reduce postoperative pain. Another indication of local anesthetic use is to reduce autonomic nervous system response to direct laryngoscopy and endotracheal intubation.^[10]^

Recent studies suggest systemic anti-inflammatory effect of local anesthetics in craniotomy patients, but also acknowledge the need for further investigation.^[19,20]^ Lidocaine has proven anti-inflammatory local and systemic effect, even at low plasma concentrations.

Therefore, we propose a clinical trial to investigate effect of lidocaine administration on perioperative levels of pro-inflammatory cytokines, which might be crucial in pathology of cerebral aneurysms. Measuring concentrations of IL-1β, IL-6, and TNF-α in plasma and CSF might provide insight regarding the effect of lidocaine on local and systemic inflammatory response.

Furthermore, this trial will investigate potential impact of decreased inflammatory response on major neurological and infectious complications in early postoperative period, as well as on outcome of the treatment.

Trial will test the hypothesis that lidocaine administration decreases concentrations of IL-1β, IL-6, and TNF-α in plasma and CSF in patients undergoing cerebral aneurysm surgery. Results of the trial could provide insight into influence of lidocaine on local and systemic inflammatory response in cerebrovascular surgery, and might improve future anesthesia practice and treatment outcome.

## Acknowledgments

The authors thank to Miljenko Košiček for support regarding statistical planning of this study. The authors are also grateful for all the colleagues and co-workers from the Department of Anesthesiology, Reanimatology, and Intensive Care Medicine, University Hospital Center Zagreb, Croatia and the Department of Neurosurgery, University Hospital Center Zagreb, Croatia.

## Author contributions

MM: study design, writing, data analysis and data interpretation; VS: study design, data interpretation and data acquisition; AK: study design, data interpretation and data acquisition; RL: study design, data interpretation and data acquisition; AS: final approval, study design and revisions.
